# Special Issue: Exploring Abiotic Stress in Plants—Mechanisms, Adaptations, and Mitigation Strategies

**DOI:** 10.3390/ijms27052182

**Published:** 2026-02-26

**Authors:** Tarek Alshaal

**Affiliations:** 1 Faculty of Agricultural and Food Sciences and Environmental Management, Institute of Applied Plant Biology, University of Debrecen, Böszörményi Str. 138, 4032 Debrecen, Hungary; alshaal.tarek@agr.unideb.hu or alshaal.tarek.ali.ahmed.ibrahim@sze.hu or tarek.ibrahim@agr.kfs.edu.eg; 2 Department of Food Biotechnology, Albert Kazmer Mosonmagyarovar Faculty, Széchenyi István University, Egyetem Sqr. 1, 9026 Győr, Hungary; 3 Soil and Water Department, Faculty of Agriculture, University of Kafrelsheikh, Kafr El-Sheikh 33516, Egypt

Abiotic stress remains one of the biggest obstacles globally to plant growth, productivity, and ecological stability. Understanding how plants sense, integrate, and respond to non-biological stress is a fundamental scientific and societal challenge due to the rapid intensification of the agricultural sector, soil degradation, climate change, and environmental pollution. The *International Journal of Molecular Sciences* Special Issue on “Exploring Abiotic Stress in Plants: Mechanisms, Adaptations, and Mitigation Strategies” offers a thorough and integrated forum for current developments in this rapidly developing topic.

Together, the 10 papers in this collection cover a wide range of topics addressing abiotic stress biology, including wounding, heat, salinity, drought, heavy metal toxicity, nutritional imbalance, and xenobiotic exposure. They cover subjects including whole-plant physiology, metabolomics, agronomic interventions, molecular regulation, and phosphoproteomics. Collectively, these investigations highlight the intricacy of stress reactions, while also pointing to viable paths toward reductions via genetic engineering, metabolic engineering, and creative agronomic techniques.

The goal of plant abiotic stress research is to fundamentally understand how sessile organisms preserve homeostasis in dynamic, frequently hostile environments. Plants use complex sensor systems, signaling cascades, and the adaptive reprogramming of metabolism and development since they are unable to avoid stress. This systems-level viewpoint is reflected in the Special Issue, which emphasizes the interconnection of metabolic plasticity, nutritional signaling, transcriptional regulation, redox biology, and epigenetic and post-translational alterations.

One of the most urgent environmental issues is heavy metal stress, especially in soils that have been affected by industrialization and intensive farming. Toxic levels of cadmium (Cd) and copper (Cu), which frequently co-occur in contaminated areas, interfere with photosynthesis, disturb cellular redox balance, and prevent root growth. The potential of folcisteine (NATCA) to promote root development and maize seed germination under combined copper–cadmium stress is examined by Dong et al. [contribution 1]. Their research highlights the critical function of redox buffering mechanisms in preserving cellular integrity under metal-induced oxidative stress by showing that ascorbate–glutathione (AsA–GSH) cycle regulation is essential for stress reduction. Folcisteine is a promising exogenous regulator that can improve early developmental resilience by maintaining glutathione homeostasis and increasing antioxidant enzyme activity.

Marques et al. [contribution 2] investigate phytochelatins as important molecular agents in cadmium mitigation, which supports this physiological viewpoint. By chelating heavy metals and facilitating their sequestration into vacuoles, phytochelatins—which are enzymatically generated from glutathione—reduce cytosolic toxicity. The authors describe genetic approaches for modifying phytochelatin pathways in order to enhance functional tolerance. A forward-looking review of phosphoproteomics in plants exposed to cadmium is provided by Marques et al. [contribution 3], who highlight how post-translational phosphorylation processes fine-tune stress signaling networks. Together, these two papers highlight the importance of combining biochemical and proteomic investigations for understanding stress adaptation by bridging traditional detoxifying systems and state-of-the-art omics techniques.

Other abiotic stimuli, like heat, salinity, and drought, challenge plants through ion imbalance, membrane instability, and water deficiency, while heavy metals mainly cause oxidative damage and metabolic interference, upsetting cellular homeostasis. Tang et al. [contribution 4] provide a thorough examination of maize’s resistance to heat stress and drought, emphasizing hormonal interactions, transcriptional reprogramming, and physiological adaptations. Their observations support the idea that coupled stresses often result in non-additive effects, the authors calling for integrative research that takes outdoor conditions into account rather than relying solely on single-stressor lab models.

Similarly, Jia et al. [contribution 5] show that tomato tolerance to salt stress and drought is improved by overexpressing the transcription factor SlPLATZ17. This study serves as an example of how osmotic adjustment, antioxidant defense, and stress-responsive gene expression can all be coordinated through the focused manipulation of regulatory genes. These works connect molecular mechanisms to crop enhancement tactics, thereby advancing translational research.

Plant stress responses are further modulated by fertilization techniques and nutrient availability. Vukmirović et al. [contribution 6] examine the impact of phosphorus fertilization on the antioxidant responses of drought-stressed common beech and sessile oak provenances. Their findings highlight the significance of provenance selection and nutrient management in forestry and ecosystem resilience, showing genotype-specific relationships between oxidative metabolism and nutrient status. Optimizing the nutrient supply in agricultural systems can improve adaptive capacity and mitigate the effects of stress.

In order to understand the mechanisms behind phenolic compound increases in *Lonicera japonica* caused by nitrate nitrogen supply, Cao et al. [contribution 7] combine metabolomics and photosynthetic study. Phenolic chemicals have two functions: they are both signaling molecules and antioxidants. This work demonstrates how nutritional regimes influence metabolic plasticity and the generation of bioactive compounds by connecting nitrogen feeding to secondary metabolism and photosynthetic performance. These results are especially pertinent to high-value crops and medicinal plants, since stress-responsive metabolites are tightly linked to qualitative features.

A growing aspect of plant stress biology is anthropogenic chemical exposure, which extends the concept of abiotic stress beyond environmental extremes. According to Zhang et al. [contribution 8], nitrogen-doped carbon dots can reduce tomato pesticide toxicity by controlling the antioxidant system. This novel technique based on nanotechnology raises new concerns about environmental safety and long-term ecological effects, while demonstrating the potential of designed nanomaterials to reduce chemical stress. It also serves as an example of how interdisciplinary approaches, such as combining plant physiology with nanoscience, can produce useful mitigation techniques.

Even though it is frequently linked to biotic interactions, mechanical damage is a type of abiotic stress that causes significant metabolic changes. In their study of metabolic reactions in injured Hippeastrum bulb scales, Wiczkowski et al. [contribution 9] find dynamic changes in primary and secondary metabolism. The intersections of wound-induced metabolic remodeling with defense priming, oxidative signaling, and resource allocation highlight the common molecular mechanisms underlying various stress responses.

The rearrangement of primary carbon metabolism is at the heart of many of these investigations. Hu et al. [contribution 10] investigate the regulating function of phosphoenolpyruvate (PEP) and its associated metabolites in plant growth and development. PEP links glycolysis, the shikimate pathway, and biosynthetic fluxes, making it a crucial metabolic node. Changes in PEP availability and utilization under stress can affect secondary metabolite synthesis, energy balance, and general growth dynamics. This contribution offers a conceptual link between stress adaptation and metabolic management by placing PEP into a larger regulatory network.

A number of recurrent themes emerge throughout this Special Issue.

First, the unifying principle of abiotic stress tolerance is redox homeostasis. Plants constantly activate antioxidant systems to limit reactive oxygen species (ROS) in response to many stressors, including heavy metals, drought, salt, pesticide exposure, and wounding. Superoxide dismutase, catalase, peroxidases, the AsA–GSH cycle, and non-enzymatic antioxidants are frequently mentioned as key players. However, ROS serve as signaling molecules in addition to having harmful consequences. Stress recognition and subsequent transcriptional activation are supported by the delicate balance between ROS production and scavenging.

Second, adaptive plasticity is orchestrated by transcriptional and post-translational regulation. The rapid reprogramming of signaling networks is shown in phosphoproteomic studies, and gene expression landscapes are coordinated by transcription factors such SlPLATZ17. Plants can dynamically integrate external stimuli with their internal metabolic status because of these regulatory layers.

Third, metabolic flexibility is both the result of and a factor in stress adaptation. The re-allocation of resources toward survival is reflected in changes in phenolic biosynthesis, carbon allocation, nitrogen assimilation, and key intermediates like PEP. The significance of systems-level techniques is demonstrated through the integration of metabolomics with physiological measures, as shown in multiple contributions.

Fourth, mitigation techniques are becoming more complex. These consist of new nanomaterials, the genetic engineering of detoxifying and regulatory mechanisms, the exogenous administration of protective chemicals (such as folcisteine), and optimized fertilization regimens. Crucially, these tactics are based on mechanical knowledge rather than empirical experimentation alone.

The urgent need to ensure crop yield and ecosystem stability in the face of global environmental change forms the larger backdrop of this Special Issue. According to climate projections, heatwaves, extended droughts, and soil salinization are becoming more frequent. Heavy metal buildup and chemical residues in soils are concurrently caused by industrialization and intensive agriculture. Water scarcity and nutrient imbalances affect forest ecosystems, and high-value horticultural and medicinal crops need to be preserved under adverse conditions.

Molecular biology, biochemistry, physiology, ecology, agronomy, and biotechnology must all be integrated to address these problems. The strength of such an integration is demonstrated by the contributions in this volume. Additionally, these articles emphasize the significance of model and crop species diversity, ranging from medicinal plants and forest trees to maize and tomatoes. Breeding and management techniques require an understanding of the intrinsic species and genotype specificity of stress tolerance.

Crucially, this Special Issue demonstrates that reactions to abiotic stress are rarely isolated events. Sequential and combined stresses are the rule rather than the exception. Future studies must use multifactorial designs more often and make use of computational modeling, sophisticated phenotyping platforms, and high-throughput omics technologies. Predictive frameworks for resilience will be made possible by integrating transcriptome, proteomic, metabolomic, and phenomic data.

The goal of translational applications is still crucial. Real-world impact is determined by agronomic viability and field validation, although molecular discoveries serve as the basis for this. Translating mechanistic insights into resilient cultivars and sustainable management techniques will require cooperation between academic researchers, breeders, agronomists, and policymakers.

As this Special Issue’s Guest Editor, I want to express my sincere gratitude to every one of the contributing authors for their inventive and hard work, as well as to the reviewers for their helpful criticism. The contributions’ richness and breadth demonstrate how vibrant the field of abiotic stress research is. This collection aims to serve as a resource for experts and a gateway for researchers wishing to delve into the intricacies of plant stress biology by combining mechanistic insights and practical viewpoints.

The goal of turning this Special Issue into a printed book is to present a coherent story that places each study inside a single conceptual framework. The book charts paths for further research while capturing a point in time in a field that is developing quickly. It emphasizes how important findings in signaling, metabolism, and redox biology can guide workable answers to environmental and agricultural problems.

Ultimately, the more general objectives of food security, environmental sustainability, and climate resilience are inextricably linked to comprehending and reducing abiotic stress in plants. Terrestrial life and human sustenance are based on plants. Improving their resilience to environmental stress is a scientific challenge as well as a social obligation. The contributions gathered here are significant steps in the direction of that goal.

